# Supplementary figures and images for: Fibre and extracellular matrix contributions to passive forces in human skeletal muscles: An experimental based constitutive law for numerical modelling of the passive element in the classical Hill-type three element model
Source: PLoS One. 2019 Nov 5;14(11):e0224232. doi: 10.1371/journal.pone.0224232 (PMC6830811; doi:10.1371/journal.pone.0224232)

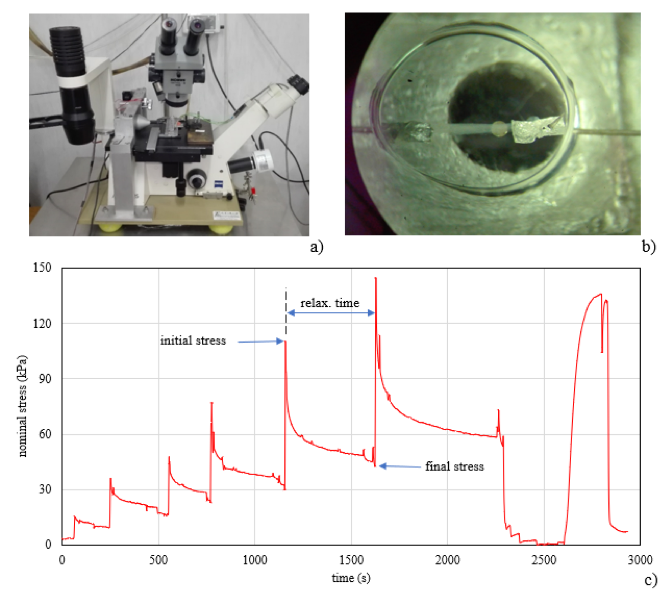

Supplement: S1 Fig — (a). Detail of a fibre bundle mounted with T-clips at its ends (b). Typical stress vs. time experimental data obtained from tests on bundles (c). (TIF) [file pone.0224232.s001.tif]

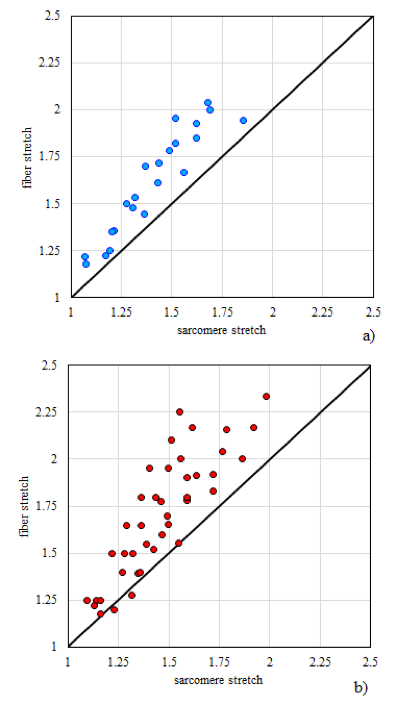

Supplement: S2 Fig — Stretch measurements on single fibres obtained by evaluating length of whole fibre (fibre stretch) and average length of sarcomeres (sarcomere stretch). The straight lines represent equal values for the two measurements. It is noted that the stretch obtained by considering the length of the whole fibre is generally higher than the stretch pertaining to sarcomeres. This could be explained by a limited compression and damage in the region of the samples held by the T-clips and, therefore, by a non-uniform strain field induced on fibres and bundles. Then, all the measures considered in the following were taken by considering the sarcomere stretch. From experimental data, the fibre stretch is generally higher than sarcomere stretch both for fast fibres (a) and slow fibres (b). (TIF) [file pone.0224232.s002.tif]

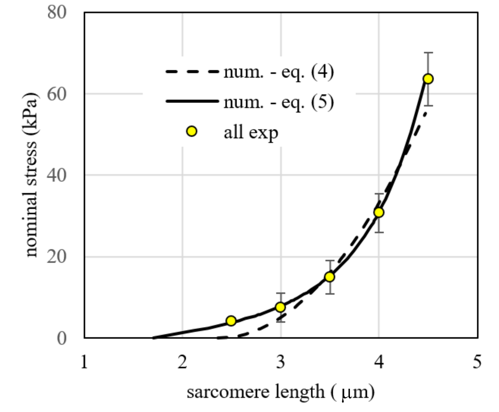

Supplement: S3 Fig — Stress vs. sarcomere length experimental data of pooled fast and slow fibres compared to numerical results obtained by the fitting equations proposed in the literature (see Eqs (4) and (5) in the main text of the present work). (TIF) [file pone.0224232.s003.tif]
