# Supplementary material for: Fibre and extracellular matrix contributions to passive forces in human skeletal muscles: An experimental based constitutive law for numerical modelling of the passive element in the classical Hill-type three element model
Source: PLoS One. 2019 Nov 5;14(11):e0224232. doi: 10.1371/journal.pone.0224232 (PMC6830811; doi:10.1371/journal.pone.0224232)
Supplement: Attachment — Submitted filename: Response to the Reviewer.docx [file pone.0224232.s005.docx]

We would like to thank you the reviewers for taking the time and effort in reviewing our manuscript. The manuscript has been revised to clarify and address all the reviewers' concerns and comments. The point by point responses are provided below.

---------------------------------------

**Response to Reviewers # 1**

Reviewer := black font

Authors := red font

**Reviewer** Authors present an experimental study on how the skeletal muscle fiber and the extracellular matrix contribute to the passive behaviour of the tissue. The study is conducted on human samples which is very interesting due to the lack of data in the literature nowadays.

**REPLY** Thank you.

**Reviewer** In spite of that, the way the paper is oriented does not provide enough and proper information for researches in the field of numerical modelling as aimed.
The title includes that numerical modelling has been developed but no results are presented. Also, Finite Element appears in the keywords but only Fig. 1 shows a finite element mesh that is not used later.

**REPLY** It is true that in this work we did not apply our results to a finite element model. However, the scope of the present work is to improve the definition of the constitutive law for the passive element in the classical Hill-type three element model often used in the FE muscle modelling. We modified the title and correspondingly rephrased some sentences in the main text to make this point clearer. In particular in the discussion we resumed the results of interest for the FE modeling with the following sentence: “*The physiological stress-strain curve for the PE element (fiber related components) in a FE model can then be described by equation (2) with parameters A = 6.93 kPa and L_0_ = 2.44 μm. The physiological characterization of the deviatoric part of the stress tensor related to ECM component can be described by equation (7) with parameters β = 168.55 MPa and L_0b_ = 1.60 μm.”.* Therefore, we believe that our results can be easily used by researcher in the field of numerical modelling and that the keyword could be kept reaching their interest.

**Reviewer** Some comments:

**Abstract**

Line 37: “However, the relative contribution of these two components in transmitting the passive forces is still under debate.” Which components? Active and passive?

**REPLY** We referred to the intra-sarcomere and extra-sarcomere (ECM) components. We made the sentence clearer. Thank you for the suggestion.

**Reviewer** Line 45: “…a stress-strain curve which were…” Replace “were” with “was”

**REPLY** Thanks for noting the incongruence. We modified with “Both were progressively elongated to obtain two stress-strain curves which were fitted to exponential equations.”

**Reviewer** **Introduction**

Line 103-105: “Considering the few data present in literature on human subjects, we decided to test muscle form elder people in this work to increase the amount of force sustained by this component.” This sentence sounds strange to me, please rewrite.

**REPLY** We modified the sentence in “Experimental data on mice showed that the ECM is stiffer in old subject than in young ones [20], therefore we decided to test muscle form elder people to maximize the observed force sustained by this component.”

**Reviewer** **Materials and methods**

Experimental testing of muscle fibers and bundles

Line 183: Is there a technical reason why the fiber/bundle was measured stepwise? Are authors aware that this is not a proper response of the tissue for modelling purposes apart from viscoelasticity?

**REPLY** We followed a protocol previously proposed in literature to separate the elastic component from the viscoelastic one. We mention this introducing the sentence: “To analyse the elastic component of the force, separated from the viscoelastic component, we followed a previously proposed protocol [14,20], and, at each step, the passive force, after a viscous recovery, as well as the cross-section and the length of the sarcomeres in the central region were recorded. Differently from previous works, which stop the relaxation after about 120 seconds overestimating the elastic tension of about 10% [14], we waited until the viscous recovery was almost complete, which lasted in some case up to 600 s, (**Figure S1c**)”

**Reviewer** Line 185: Authors should mention here that the fiber recovers its initial length before isometric activation.

**REPLY** Done

**Reviewer** Constitutive modelling of muscle fibers

It seems that, according to what is explained in the text, all the passive contribution in the muscle fiber is due to the Parallel Element (PE). Is this correct? What happened with the Elastic Series Element (SE)? The total passive force is the sum of both the PE and SE, are authors neglecting the SE? Where is located the titin in the scheme of the constitutive model in Fig. 1?

**REPLY** In the Hill’s three-element model the SE is in series with the contractile element CE as shown in Fig. 1. CE is supposed to extend (or shorten) freely in the passive state of the muscle and all the tension is sustained by the PE (in the fiber) and the ECM if included in the model. Since we are dealing with a passive muscle, we did not attribute any component to the SE here. However, we have characterized it in our previous paper cited in the text (Marcucci et al. BMMB 2017).

**Reviewer** Lines 217-218: “Applying the derivative (3), the initial tangential stiffness for this formulation is null.” Something is missing, the initial slope of eq (4) (lambda_fib = 1) is 8*P_0

**REPLY** A typo was present in equation (4), giving rise to the incongruence. In the revised version the equation is corrected according to the bibliographic reference, corresponding to a null initial slope.

**Reviewer** Line 260: Remove one “definition”

**REPLY** Done

**Reviewer** Constitutive modelling of muscle fiber bundles

Eq. (6) The dyadic product should only include the orientation vector in the reference configuration, otherwise is nonsense.
This equation represents an atypical way to define the behaviour of the muscle tissue in contrast to the derivation of the constitutive relationship from a strain energy density function.

**REPLY** We cannot agree with the Reviewer. The first Piola-Kirchhoff stress tensor **P** we adopt in the paper is a so-called two-point tensor, with the first index related to the deformed configuration and the second one to the undeformed configuration. Accordingly, its energy conjugated strain measure is the deformation gradient, which is another two-point tensor. The constitutive formulation could be similarly defined by using the second Piola-Kirchhoff stress tensor. In the latter case each stress component is related to a diadic product based on the unit vectors (both) in the reference configuration, as proposed by the Reviewer.

We have reformulated the equation by using only terms related to stress, without mixing terms of stress and energy derivatives. The stress can be derived by a corresponding strain energy function but, for the purpose of its implementation, is more useful to have its direct formulation.

**Reviewer** **Results**

Muscle fibers

Lines 289-301: According to my previous comment, it would be surprising that with an extension (passive) test, authors could determine the maximum isometric stress. The conclusion here is that more parameters are needed to fit the passive response of the tissue.
“…in our approach the active tension values are not used to define the passive tension behaviour and a single multiplicative, stress like parameter is identified.” So, why then P_0 is still in eq. (5)?

**REPLY** We agree with the Reviewer that analyzing the tissue in passive condition is not possible to deduce any information about the maximum isometric stress. Equations (4) and (5) describe constitutive formulations that are usually adopted in the literature and, in our opinion, are contradictory just for the use of the maximum isometric stress as a parameter. We used these only for comparative results with our approach, where we do not mix passive and active behavior.

**Reviewer** The overall paragraph is confusing if eq. (4) and (5) are not used in the rest of the work and the results discussed are in the form of supplementary material.

**REPLY** Equations 4 and 5 are introduced only for a comparison of our approach with constitutive equations of the PE previously proposed in literature. We hope that the previous description and the new version of the manuscript with most of the supplementary information in the main text have clarified the paragraph

**Reviewer** Muscle bundles

Fig.3: The data showed in this form is confusing, please connect the points that belong to the same sample.

**REPLY** We agree that the previous version fo the figure was somehow confusing. We connected all the points that belong to the same sample, with a line for each sample, but, in our opinion, this approach hides the main message of the figure. We decided to add a different color for each sample keeping separated the main colors for fibers and bundles.

**Reviewer** On the other hand, sarcomere length is different for each sample for both fibers and bundles, why? Are values obtained in Tab. 2 and Tab. 3 interpolated?

**REPLY** We confirm that experimental data of sarcomere length are in general different for both fibres and bundles, because it is different the initial length of them and their incremental elongation. Other works controlled the total length of the fiber and proposed to correlate it to the sarcomere length. However, we preferred to record the real average sarcomere lengths after each elongation. This approach is more precise but leads to a dispersion in sarcomere lengths. In order to have an average response of the different groups (fast and slow fibers, bundles) a multiple fitting has been made considering all the data of each group and obtaining the corresponding stress-elongation curves. The paragraph concerning this aspect has been slightly modified to make clearer the fitting method adopted.

**Reviewer** Line 316: Why using the volume fractions? They do not appear in eq. (8) and how was the fitting performed if eq. (8) represents the deformation energy? Is eq. (9) perhaps?

**REPLY** Yes, the referenced equation number was wrong. With the new version of the paper the equations referred to the strain energy function were all omitted. The wrong reference has been corrected.

**Reviewer** **Discussion**

Lines 396-398: This last sentence is illegible.

**REPLY** We have eliminated the sentence which was not necessary.

**Reviewer** Lines 411: “Here we focused only in the exponential part, considering it the most meaningful for the FE modelling, the main scope of this work” This sentence has to be further explained and proved. Does the muscle physiologically undergoes such levels of deformation (up to 200%)?

**REPLY** The range of sarcomere length, as measured in vivo with microendoscopy in vastus lateralis by Chen et al 2018 is approximately 2.8-3.2 um, changing in proportion to the angle between leg and thigh. Similar values (2.84-3.48 um) have been measured in soleus muscles (Chen and Delp 2016) and also in tibialis anterior (Lichtwark et al 2018) in relation to variations of the ankle angle. The range of measured sarcomere lengths should be seen inside the theoretical whole range of sarcomere length from slack length close to 2.2-2.4 um to 4.0-4.2 um as the end of the overlap between thick filaments (1.6 um long in humans as in all vertebrates) and thin filament (1.2-1.3 um long in humans, see Lieber et al 1994). Thus the potential range of elongation is around 180% of slack length. The sarcomere length we explored in our experiments in vitro covers the whole range from 2.4 to 4.5 um. In the revised version of the manuscript the issue about the physiological range of sarcomere length is discussed at page 24

Chen X, Sanchez GN, Schnitzer MJ, Delp SL. Changes in sarcomere lengths of the

human vastus lateralis muscle with knee flexion measured using in vivo

microendoscopy. J Biomech. 2016 Sep 6;49(13):2989-2994. doi:

10.1016/j.jbiomech.2016.07.013. Epub 2016 Jul 22. PubMed PMID: 27481293; PubMed

Central PMCID: PMC5507365.

Chen X, Delp SL. Human soleus sarcomere lengths measured using in vivo microendoscopy at two ankle flexion angles.

J Biomech. 2016 Dec 8; 49(16):4164-4167. Epub 2016 Nov 10.

Lichtwark GA, Farris DJ, Chen X, Hodges PW, Delp SL. Microendoscopy reveals

positive correlation in multiscale length changes and variable sarcomere lengths

across different regions of human muscle. J Appl Physiol (1985). 2018 Sep 13.

doi: 10.1152/japplphysiol.00480.2018. [Epub ahead of print] PubMed PMID:

30212307.

Lieber RL, Loren GJ, Fridén J

In vivo measurement of human wrist extensor muscle sarcomere length changes.

J Neurophysiol. 1994 Mar;71(3):874-81.

DOI: 10.1152/jn.1994.71.3.874 PMID: 8201427 [Indexed for MEDLINE]

We have eliminated the sentence mentioned by the referee and introduced the following:

“Recent studies carried out with microendoscopy on human vastus lateralis in vivo showed a working range between 2.8 and 3.2 um with knee angle changing only from 50° to 110° (Chen et al 2018). This represents only part of the potential whole range of sarcomere length from slack length close to 2.2-2.4 um to 4.0-4.2 um as the end of the overlap between thick filaments (1.6 um long in humans as in all vertebrates) and thin filament (1.2-1.3 um long in humans, see Lieber et al 1994). The sarcomere lengths explored in this study covered the entire potential range.”

**Reviewer** I disagree that the main scope of the work is FE modelling, the results and conclusions are oriented to compare passive properties of ECM and fibers.

As mentioned in the first point, we have modified the text to explain how the results can be of some help in the FE modelling.

---------------------------------------

**Response to Reviewers # 2**

Reviewer := black and blue font

Authors := green font

**Reviewer #2**: What are the main claims of the paper and how significant are they for the discipline?
The paper claims that passive tension generated by the vastus lateralis of aged humans comes primarily from the extracellular matrix, rather than intracellular proteins, as evidenced by studies of passive tension produced by single muscle fibers and bundles of muscle fibers. Additionally, the paper claims that the finite element model presented represents a significant improvement on past finite element models of passively stretched muscle, largely because it more accurately represents the physiological relationships of the parallel, series, and contractile elements typically included in Hill-type models of muscle physiology.
The determinants of passive force in stretched skeletal muscle remain under debate. This study provides one example of a muscle in which the extracellular matrix dominates the passive mechanical properties of the tissue. Such studies can provide insight into the contributions of different components of muscle to passive muscle mechanics and, when viewed in the aggregate with similar studies, will provide insight into variation in the determinants of passive muscle mechanics across vertebrates.

**REPLY** We would like to thank the reviewer for the attention given to the manuscript and for highlighting its main results.

**Reviewer** Are the claims properly placed in the context of the previous literature? Have the authors treated the literature fairly?
Yes. A good overview of literature exploring the sources of passive tension in muscle is provided. Evidence is provided for both the extracellular matrix and the intracellular proteins as the primary contributor to passive tension in muscle, and this accurately represents the state of knowledge within the field of muscle physiology. A description of the use of finite element models to study muscle is also included.

**REPLY** Thank you.

**Reviewer** Do the data and analyses fully support the claims? If not, what other evidence is required?
Yes. The higher stress generated by fiber bundles relative to single muscle fibers at the same sarcomere length indicate that the extracellular matrix material present in fiber bundles is responsible for most of the passive tension generated.

**REPLY** We thank the reviewer for noting this result of the manuscript.

**Reviewer** Do any methods warrant publication of protocols or algorithms as supplementary material? If a protocol is provided, are there any important deviations from it?
No. In its current form, the paper details many important methods of the study in the included supplemental document, but I see no reason why these methods could not be included in the main body of the paper. Moving the methods sections included in the supplemental material into the main body of the manuscript would improve the readability of the paper.

**REPLY** We thank the reviewer for this useful suggestion. Since the journal has no prescription about the text length, we decided to fully accomplish the reviewer request and move all the methods information from the supplementary material to the “Materials and Methods” section in the main text. We agree that this modification improved the readability of the manuscript.

**Reviewer** If the paper is unsuitable for pub in its present form, does the study show significant potential that it should be resubmitted?
The paper shows significant potential. The main barriers to publication are stylistic. There are enough errors in grammar and punctuation that they became distracting, and sometimes make the content difficult to understand. There also appear to be some residual edits that were not removed by the authors in the main body of the manuscript. Words that have been struck through with a text editor remain. Use of a professional editing service, such as <https://authorservices.springernature.com/language-editing/> could help remedy these issues and improve the readability of the paper.

**REPLY** To accomplish .an editorial staff request, the text has been revised by a mother-tongue colleague, both in the main text and in the supplementary data now included in the main text. We hope that the deep revision has improved the style of the manuscript to the required level for the publication.

**Reviewer** Additionally, the graphic depicted in figure 1 is not described in sufficient detail and figure 5 contains an unlabeled trace. Finally, as stated above, the methods presented in the supplemental material should be included in the main body of the text to improve readability and allow the manuscript to stand alone.
While these stylistic and organizational concerns reduce the readability of the paper, the question asked by the paper, the experimental methods, and the analytical approach are all strong, and warrant publication.

**REPLY** We are happy that the reviewer recognized the importance of the paper. We have moved the whole supplementary information about the methods in the main text and modified the captions of figures 1 and 5 as required. The graphic problem of figure 5 has been amended.

**Reviewer** Are data deposited in appropriate repositories?
It is stated that all data are fully available without restriction, but a repository used is not specified.

**REPLY** We have added the raw data in a supporting information file

**Reviewer** Are methods details sufficient for reproduction?
Yes, but only when the supplemental data files are considered. The methods section from the supplemental data file should be merged with the main body of the manuscript so that the paper can stand alone without the supplemental material.

**REPLY** We moved the supplementary material into the main text as requested

**Reviewer** Is the manuscript organized and written clearly enough to be accessible to non-specialists?
Yes. The organization of the paper was logical. Addressing grammatical concerns at the sentence level will improve readability.

**REPLY** A mother tongue colleague edited the manuscript and several improvements were made for the readability as described.

**Reviewer** Specific comments:

**Abstract:**

Page 3 Line 37: The abstract refers to the relative contributions of two components in transmitting passive force, but it is not clear which two components are being referenced. Three components are listed immediately before this statement: intra-sarcomeric proteins, fiber cytoskeleton, and ECM.

**REPLY** We have modified the sentence as requested also by the first reviewer in “However, the relative contribution of the intra- and extra- sarcomeric components in transmitting the passive forces is still under debate”

**Reviewer** Introduction:

Page 6 line 111: the contractile element is influenced by a length-tension relationship as well, why is only the force-velocity relationship referenced here?

**REPLY** We agree. We have added this component.

**Reviewer** Page 7 line 144: Could the caption for figure 1 include a description of what the graphic is depicting, how the graphic was generated, and what specifically the arrows are pointing to? What are the grid-like lines overlaying the extracellular matrix depicting?

**REPLY** The legend has been modified to account for the request of the Reviewer.

**Reviewer** Page 8 lines 146-150: These sentences seem like a summary of results and potential significance. Would these be better suited to the results or discussion section?

**REPLY** We understand the reviewer comment, however we would like to give to the general reader a brief description of the work aims and results before going through the results and discussion. Therefore, we would like to leave this sentence at his place.

**Reviewer** Materials and Methods:

Page 8 line 162: If the epimysium is included, were the samples taken at the muscle perimeter? Do you mean perimysium here?

**REPLY** Yes, we thank the reviewer for noting it. The word has been corrected.

**Reviewer** Page 8 line 163: Could you elaborate on how fibers were dissected? How was fiber damage avoided?

**REPLY**: more info about the dissection and mounting are given in the new version of the Methods which has been incorporated in the main body of the paper (pages 8-9).

**Reviewer** Page 8 line 169: This is the first mention of T-shaped clips. Could you define them for readers that aren’t familiar with this technique?

**REPLY** We have included the following sentence “T-shaped aluminium clips (T-clips) were mounted at each end of the fibers or fiber bundles leaving 1-2 mm free and their sides were folded tightly around the fiber or the bundle. Each clip was provided by a small hole so the fiber or bundle could then be transferred to the apparatus (**Figure S1a**) and mounted horizontally between two hooks: one linked to the puller (SI, Heidelberg, Germany) to control the length of the specimen and the other to the force transducer (AME-801 SensorOne, Sausalito, California) (**Figure S1b**).”

**Reviewer** In general, it seems like a lot of the relevant methods details have been relegated to the supplemental material. Is there a reason that information on the experimental setup, cryo-sectioning and staining approach, and statistical analysis is not included in the main document?

**REPLY** We moved all the required information in the main text as described before.

**Reviewer** From the methods it is not entirely clear why slow and fast twitch fibers needed to be distinguished. Do these display distinct mechanical properties, and can you provide a citation indicating this?

**REPLY** The need to take into account the "type of fiber", i.e. the presence of specific and distinct myosin isoforms is determined by the evidence which, although partially controversial, show that passive mechanical properties of single fibers are different depending on the "fiber type", Titin isoforms, which are likely the major determinants of single fiber passive tension, have different molecular weight and different length. Longer titin isoforms are present in fibers with higher compliance and are present in fibers expressing a slow myosin isoform (slow fibers). The issue is taken up with some detail in the discussion at pages 24-25.

**Reviewer** **Results:**

Line 327: What is the yellow trace on figure 5? I can assume it is stress from individual fibers, but this is not indicated in the figure legend or caption.

**REPLY** Figure 5 and legend have been corrected.


**Reviewer** **Discussion:**

Page 18 line 362 “It likely depends on muscle type, species and aging” can you provide citations for these statements?

**REPLY** We have included the required citations

**Reviewer** Figure 7: Surpaspinatus is misspelled in the figure legend

**REPLY** The legend of figure 7 has been completely rewritten.

6. PLOS authors have the option to publish the peer review history of their article ([what does this mean?](https://journals.plos.org/plosone/s/editorial-and-peer-review-process#loc-peer-review-history)). If published, this will include your full peer review and any attached files.


If you choose “no”, your identity will remain anonymous but your review may still be made public.


**Do you want your identity to be public for this peer review?** For information about this choice, including consent withdrawal, please see our [Privacy Policy](https://www.plos.org/privacy-policy).

Reviewer #1: No

Reviewer #2: No


[NOTE: If reviewer comments were submitted as an attachment file, they will be attached to this email and accessible via the submission site. Please log into your account, locate the manuscript record, and check for the action link "View Attachments". If this link does not appear, there are no attachment files to be viewed.]


While revising your submission, please upload your figure files to the Preflight Analysis and Conversion Engine (PACE) digital diagnostic tool, <https://pacev2.apexcovantage.com/>. PACE helps ensure that figures meet PLOS requirements. To use PACE, you must first register as a user. Registration is free. Then, login and navigate to the UPLOAD tab, where you will find detailed instructions on how to use the tool. If you encounter any issues or have any questions when using PACE, please email us at [figures@plos.org](mailto:figures@plos.org). Please note that Supporting Information files do not need this step.

**Journal requirements:**

When submitting your revision, we need you to address these additional requirements.

1. Please ensure that your manuscript meets PLOS ONE's style requirements, including those for file naming. The PLOS ONE style templates can be found at
<http://www.journals.plos.org/plosone/s/file?id=wjVg/PLOSOne_formatting_sample_main_body.pdf> and <http://www.journals.plos.org/plosone/s/file?id=ba62/PLOSOne_formatting_sample_title_authors_affiliations.pdf>

**DONE**

2. Thank you for stating the following financial disclosure:
 "The funders had no role in study design, data collection and analysis, decision to publish, or preparation of the manuscript."

**Please, include the following statement: “The authors received no specific funding for this work.”**

3. Please include captions for your Supporting Information files at the end of your manuscript, and update any in-text citations to match accordingly. Please see our Supporting Information guidelines for more information: <http://journals.plos.org/plosone/s/supporting-information>.

**DONE**
